# Characterization of pain-related mechanisms and bone destruction in mild and severe monoiodoacetate-induced hip arthropathy in rats

**DOI:** 10.1186/s12891-026-10235-y

**Published:** 2026-07-18

**Authors:** Yasuhiro Furihata, Yuya Kawarai, Ikuko Tajiri, Kazuhide Inage, Miyako Suzuki-Narita, Rui Hirasawa, Shigeo Hagiwara, Junichi Nakamura, Jun-ichiro Ikeda, Seiji Ohtori

**Affiliations:** 1 Department of Orthopaedic Surgery, Graduate School of Medicine, Chiba University, 1-8-1 Inohana, Chuo-ku, Chiba City, Chiba 260-8677 Japan; 2 Department of Bioenvironmental Medicine, Graduate School of Medicine, Chiba University, 1-8-1 Inohana, Chuo-ku, Chiba City, Chiba 260-8677 Japan; 3 Department of Diagnostic Pathology, Graduate School of Medicine, Chiba University, 1-8- 1 Inohana, Chuo-ku, Chiba City, Chiba 260-8677 Japan

**Keywords:** Rapidly destructive coxarthrosis, Monoiodoacetate, MIA-induced hip arthropathy, Pain-related behavior, Spinal microglia, Bone resorption

## Abstract

**Background:**

This study aimed to characterize pain-related mechanisms and the bone-destructive environment in mild and severe monoiodoacetate (MIA)-induced hip arthropathy in rats, with particular focus on features relevant to rapidly destructive coxarthrosis.

**Methods:**

Rats were divided into sham, low-dose MIA, and high-dose MIA groups (*n* = 12 per group) and received intra-articular injections of saline, low-dose MIA, or high-dose MIA, respectively. Pain-related behaviors were assessed using von Frey and weight-bearing tests. Radiological and histological changes were evaluated using microcomputed tomography and the Osteoarthritis Research Society International scoring system. The proportion of calcitonin gene-related peptide (CGRP)-immunoreactive dorsal root ganglion (DRG) neurons and the number of ionized calcium-binding adaptor molecule 1 (Iba1)-immunoreactive microglia in the spinal dorsal horn were quantified immunohistochemically. Synovial mRNA expression of tumor necrosis factor-α (*Tnf-α*) and receptor activator of nuclear factor-kappa B ligand (*Rankl*), as well as osteoclast-like multinucleated cells in the femoral head, were also quantified.

**Results:**

The high-dose MIA group showed significantly greater mechanical hypersensitivity and reduced weight-bearing capacity than the low-dose MIA and sham groups. This group also demonstrated more severe joint destruction, higher OARSI scores, a higher proportion of CGRP-immunoreactive DRG neurons, and an increased number of Iba1-immunoreactive microglia in the spinal dorsal horn. Synovial *Tnf-α* and *Rankl* mRNA expression levels were significantly increased in the high-dose MIA group compared with those in the sham and low-dose MIA groups. These changes were accompanied by an increased number of osteoclast-like multinucleated cells and bone resorption cavities compatible with Howship’s lacunae in the femoral head.

**Conclusions:**

High-dose MIA-induced hip arthropathy was characterized by more severe pain-related behavior, a higher proportion of CGRP-immunoreactive DRG neurons, an increased number of Iba1-immunoreactive microglia in the spinal dorsal horn, and a more pronounced osteoclast-associated bone-destructive environment than low-dose MIA-induced hip arthropathy. These findings suggest that the high-dose MIA model exhibits several experimental features relevant to rapidly destructive hip disease.

## Background

Originally reported by Forestier in 1957 [1], rapidly destructive coxarthrosis (RDC) was later recognized as a distinct disorder by Postel et al. [2]. RDC is characterized by rapid destruction of the femoral head and/or acetabulum within 6–12 months, mostly affecting older women [3]. The reported incidence is 3.5%–15.7% [4–6]. Its major clinical manifestation is severe hip pain. Intense pain at rest, joint motion restriction, and gait disturbance further contribute to a marked decline in activities of daily living. Thus, total hip arthroplasty is required in most cases. However, these patients are typically older and have multiple comorbidities, which increase surgical risks. Furthermore, surgical procedures for RDC are generally complex, with the frequent need for adjuncts such as cages or revision implants, often leading to increased intraoperative blood loss and prolonged operative time [7–10]. Owing to both patient-related factors and the invasiveness of surgery, several patients with RDC are either unable or unwilling to undergo operative treatment. Consequently, many of them ultimately become bedridden; this issue remains a significant and unresolved challenge in clinical practice.

Currently, the mechanisms underlying bone destruction in RDC remain unclear. However, recent studies have suggested that RDC pathogenesis may involve local inflammation and osteoclast activation. Indeed, proinflammatory cytokines and cartilage-degrading enzymes in the synovial fluid and synovial tissue of patients with RDC are markedly increased [11, 12]. Furthermore, subchondral insufficiency fractures and subsequent release of bone and cartilage debris into the joint space may activate inflammasomes in intra-articular macrophages, causing intense inflammation and osteoclast-mediated bone resorption [13]. However, previous clinical studies have small sample sizes and heterogeneous patient backgrounds, including the presence of comorbidities and the use of antiresorptive agents for osteoporosis, hindering the generalization of these findings. Currently, preclinical studies using experimental models relevant to rapidly destructive hip disease are limited, with no study simultaneously examining osteoclast-associated histopathological changes and the expression of osteoclastogenic factors such as tumor necrosis factor-α (TNF-α) and receptor activator of nuclear factor-kappa B ligand (RANKL).

High-dose intra-articular injection of monoiodoacetate (MIA) into the rat hip joint has been shown to induce rapid and severe bone destruction with features resembling those observed in RDC [14]. Therefore, we used low- and high-dose MIA-induced hip arthropathy to characterize the pain-related and bone-destructive changes associated with different severities of joint destruction. We hypothesized that high-dose MIA-induced hip arthropathy would exhibit more pronounced pain-related behavior, peripheral nociceptive and spinal neuroinflammatory responses, and osteoclast-associated bone destruction than low-dose MIA-induced hip arthropathy. Specifically, we aimed (1) to characterize pain-related behavior, CGRP expression in DRG neurons, and spinal microglial responses and (2) to quantify osteoclast-related pathology, including synovial expression of *Tnf-α* and *Rankl* and the number of osteoclast-like multinucleated cells in the femoral head, among the sham, low-dose MIA, and high-dose MIA groups.

## Methods

### Animals

All experimental procedures were approved by the institutional ethics committee (Approval No. 7 − 005) and conformed to the National Institutes of Health Guidelines for the Care and Use of Laboratory Animals. This study was conducted in accordance with the ARRIVE guidelines.

We used male Sprague–Dawley rats weighing 350–400 g (CLEA, Tokyo, Japan). The required number of animals was calculated using the resource equation method [15]. A total of 36 rats were randomly assigned to three groups (*n* = 12 per group): the sham group, the low-dose MIA group, and the high-dose MIA group. The numbers of animals used for each analysis are described in the relevant Methods subsections and figure legends. These rats were housed in a semi-barrier facility under controlled environmental conditions (12-h light/dark cycle, 21–23 °C, 45%–65% humidity) with ad libitum access to standard rodent chow (CRF-1; Oriental Yeast Co., Ltd., Tokyo, Japan) and water. Pain and general condition were regularly monitored throughout the experimental period. Animals were euthanized immediately if they experienced severe or unrelieved pain or distress, as evidenced by difficulty in feeding or drinking, signs of severe suffering, persistent diarrhea, bleeding, soiling of the external genital area, or rapid and irreversible body weight loss of 20% or more. In addition, animals were euthanized without delay if their general condition was adversely affected by surgical procedures. At the end of the experimental period, or when humane endpoints were reached, rats were euthanized under deep inhalation anesthesia with sevoflurane. Briefly, rats were placed in an induction chamber and exposed to a high concentration of sevoflurane in oxygen using a precision vaporizer until loss of consciousness was achieved. Adequate depth of anesthesia was confirmed by loss of the righting reflex and absence of pedal withdrawal response to toe pinch. Sevoflurane exposure was then continued until respiratory arrest occurred and maintained for at least 1 min thereafter. Death was confirmed by the absence of heartbeat, respiration, corneal reflex, and response to toe pinch. This procedure was selected to minimize pain and distress during euthanasia and was performed in accordance with institutional animal welfare requirements.

### Intra-articular injection of MIA

Anesthesia was induced by intraperitoneal injection of medetomidine (0.3 mg/kg; Nippon Zenyaku Kogyo Co., Ltd., Tokyo, Japan), midazolam (4.0 mg/kg; Maruishi Pharmaceutical Co., Ltd., Osaka, Japan), and butorphanol (5.0 mg/kg; Meiji Seika Pharma Co., Ltd., Tokyo, Japan), in accordance with the established protocols [16]. Adequate depth of anesthesia was confirmed by loss of the righting reflex and absence of response to toe pinch before the procedure. Respiratory pattern and general condition were monitored throughout the procedure. Medetomidine, an α2-adrenoceptor agonist, and butorphanol, an opioid agonist–antagonist, were included to provide analgesia. All procedures were performed aseptically. Intra-articular injections were administered into the right hip joint posteriorly [17, 18]. Rats in the high-dose MIA group received 4 mg of MIA dissolved in 25 µL of sterile saline containing 1% Fluorogold (FG; Fluorochrome, Denver, CO, USA). Rats in the low-dose MIA group received 0.5 mg of MIA in the same vehicle, whereas rats in the sham group received 25 µL of sterile saline containing 1% FG only. The low-dose and high-dose MIA injections were used to induce mild and severe MIA-induced hip arthropathy, respectively. Injection was confined to the joint cavity as previously described [18], with the MIA dose based on earlier studies [14].

### Microcomputed tomography (µCT) scanning

Rats were placed in an induction chamber, with anesthesia maintained with oxygen mixed with 1.5% sevoflurane (Mylan Inc., Canonsburg, PA, USA) at a flow rate of 3.0 L/min. The sevoflurane concentration was gradually adjusted to achieve the desired anesthetic depth [19]. CT scans were obtained before and at 1, 2, and 4 weeks after MIA injection (*n* = 6 per group). The right hip joints were imaged using a CosmoScan FX system (Rigaku Corp., Tokyo, Japan) with 360° rotation at 90 kV and 88 µA, a field of view of 45 mm, and an exposure time of 18 s. The entire hip joint was scanned, and coronal and axial images centered on the femoral head were analyzed. Radiographic changes were graded according to the Kellgren–Lawrence (KL) classification system [20, 21].

### Histopathological analysis

Samples were obtained 28 days after MIA or saline administration. The animals were anesthetized intraperitoneally and perfused transcardially, as previously described [22]. Thereafter, tissues from the right hip joint, including bone, cartilage, synovium, and capsule, were harvested. We transected the limbs at the mid-femur and subsequently immersed the femoral head in 10% neutral buffered formalin for 3 days. Next, specimens were decalcified in K-CX solution (FALMA, Tokyo, Japan) and 5% sodium sulfate for 30 and 16 h, respectively, then embedded in paraffin for coronal sectioning. Serial sections sliced at 8 μm were stained with hematoxylin and eosin, safranin O, and toluidine blue. For histological analysis, we used the Osteoarthritis Research Society International (OARSI) scoring system [23]. For each femoral head (*n* = 6 per group), 10 sections centered on the maximal diameter were evaluated. We recorded both the depth (grade) and width (stage) of cartilage degeneration and calculated the final score by multiplying the two values. The mean score from the 10 sections was used as the representative value for each animal. Osteoclast-like multinucleated cells were counted across the entire femoral head in the same 10 sections. Multinucleated giant cells in contact with the bone surface and associated with resorption cavities, including Howship’s lacunae, were defined as osteoclast-like multinucleated cells. The total number of osteoclast-like multinucleated cells across the 10 sections was used as the representative value for each animal. Histological scoring and cell counting were performed by an observer blinded to group allocation.

### Pain behavioral tests

The von Frey test and weight-bearing test (WBT) were used to assess pain-related behavior in all rats (*n* = 12 per group). Mechanical hypersensitivity was evaluated with the von Frey test and expressed as the 50% withdrawal threshold. The ratio of weight borne on the right (affected) limb relative to the left limb was assessed using the WBT. For the von Frey assay, rats were acclimated to the testing room for 60 min and then placed in the apparatus for an additional 60 min of habituation before testing. An experimenter blinded to group allocation conducted all the measurements between 9:00 a.m. and 3:00 p.m., in accordance with previously published protocols [24]. Using the up–down method with von Frey filaments (Monofilament kit; Smith & Nephew, Germantown, WI) [25], we evaluated mechanical sensitivity of the right hind paw plantar surface at baseline and at 7, 14, and 28 days after induction. Each filament was applied for 4 s or until the rat withdrew its paw. Results are expressed as the 50% withdrawal threshold (g). Stimulus intensity ranged from 1 g to 60 g, corresponding to filament numbers 4.08, 4.17, 4.31, 4.56, 4.74, 4.93, 5.07, 5.18, 5.46, and 5.88. An incapacitance meter (IITC Life Science, Woodland Hills, CA, USA) was used for the WBT. Rats were positioned so that each hind limb rested on a separate load cell. After stable positioning, the force applied to each plate was recorded over a manually triggered 5-s interval. Each rat underwent measurement five times, and the mean value was computed. The weight-bearing ratio was then calculated as the mean weight on the affected limb divided by that on the contralateral limb, providing an index of asymmetry in weight distribution between the two hind limbs [26, 27].

### Immunohistochemistry

On day 28 after induction, samples were collected from rats in each group (*n* = 6 per group). Following transcardiac perfusion with 0.9% saline and 500 mL of 4% paraformaldehyde in phosphate-buffered saline (PBS), we harvested the ipsilateral L4 dorsal root ganglion (DRG) and lumbar spinal cord. Tissues were post-fixed overnight in 4% paraformaldehyde at 4 °C and then cryoprotected in 0.01 M PBS containing 20% sucrose for 20 h at 4 °C before liquid nitrogen freezing. DRG and spinal cord samples were cryosectioned at 10 and 30 μm, respectively (Leica CM3050S, Wetzlar, Germany), and mounted on poly-L-lysine–coated slides. After blocking the sections at room temperature for 90 min with PBS containing 0.3% Triton X-100 and 3% skim milk, we incubated them for 20 h at 4 °C with rabbit anti-CGRP (1:1000; Cat# PC205L, Chemicon, Temecula, CA, USA) or rabbit anti–ionized calcium-binding adaptor molecule 1 (Iba1) (1:1000; Cat# 019-19741, Wako, Osaka, Japan) as primary antibodies. For the secondary antibodies, Alexa 488–conjugated goat anti-rabbit IgG (for CGRP or Iba1, 1:1000; Molecular Probes, Eugene, OR, USA) was used. After each step, sections were rinsed thrice in PBS. Immunostained sections were observed using a fluorescence microscope (Olympus, Tokyo, Japan) by investigators blinded to group allocation. For DRG analysis, whole DRG sections containing FG-labeled neurons were evaluated rather than a predefined region of interest. For each animal, 10 sections centered on the section with the largest DRG area were analyzed. The number of FG-labeled CGRP-immunoreactive (ir) DRG neurons was counted in each section, and the proportion of these neurons relative to the total number of FG-labeled DRG neurons was calculated, as described previously [16, 17]. The mean proportion from the 10 sections was used as the representative value for each animal. All DRG analyses were performed by investigators blinded to group allocation. For Iba1 analysis, lumbar spinal cord sections in which the ipsilateral and contralateral dorsal horns could be evaluated comparably were selected. Iba1-immunoreactive (ir) microglia were quantified in a standardized region of interest placed in the ipsilateral superficial dorsal horn, corresponding approximately to Rexed laminae I–II. The same anatomical region was analyzed in all animals. Iba1-ir microglia within the defined region were manually counted under 400× magnification using a counting grid and expressed as the number of cells per 0.1 mm², as described previously [16, 17]. For each animal, 10 spinal cord sections were analyzed, and the mean value from these sections was used as the representative value for statistical analysis. Three experienced observers blinded to group allocation independently assessed the staining, and results were accepted only when the counts of at least two of the three observers differed by less than 5%.

### RNA extraction and quantitative real-time polymerase chain reaction (qPCR)

At 28 days following induction, synovial tissue samples were harvested from the ipsilateral hip joints of rats in each group (*n* = 6 per group). Tissues were immediately frozen in liquid nitrogen, pulverized using a mortar and pestle, and suspended in 1 mL of ISOGEN reagent (Nippon Gene, Tokyo, Japan). Samples were homogenized on ice using a pellet pestle motor (Sigma-Aldrich, St. Louis, MO, USA; Z359971). RNA was extracted using the acid guanidinium thiocyanate–phenol–chloroform method according to the manufacturer’s instructions. After adding chloroform, we centrifuged the samples for 15 min and collected the aqueous phase. RNA was precipitated with 0.8 mL of isopropanol, washed with 70% ethanol, and dissolved in nuclease-free water. Its quality and quantity were assessed using a NanoDrop 2000 spectrophotometer (Thermo Fisher Scientific, Waltham, MA, USA) and RNA Pico Chip Bioanalyzer 2100 (Agilent, Santa Clara, CA, USA), with integrity numbers ranging from 7.2 to 9.1. For cDNA synthesis, 500 ng of RNA was reverse-transcribed using the High-Capacity cDNA Reverse Transcription Kit (Applied Biosystems, Foster City, CA, USA). We conducted qPCR on a CFX Connect Real-Time PCR System (Bio-Rad, Hercules, CA, USA) using SsoAdvanced Universal SYBR Green Supermix (Bio-Rad). Table 1 lists the primer sequences. Each reaction was run twice. Relative mRNA expression was normalized to glyceraldehyde 3-phosphate dehydrogenase using the ΔΔCt method [28], with expression levels calculated as fold changes relative to the sham group.

**Table 1:** Primer pairs used for quantitative PCR analysis

Gene Name	Forward Primer	Reverse Primer
*Gapdh*	5’ CTCTCTGCTCCTCCCTGTTC3’	5’TACGGCCAAATCCGTTCACA3’
*Tnf-α*	ACTGAACTTCGGGGTGATCG	GCTTGGTGGTTTGCTACGAC
*Rankl*	CCTGTACTTTCGAGCGCAGA	GTCGAGTCCTGCAAACCTGT

### Statistical analysis

Statistical analyses were performed using GraphPad Prism version 11 (GraphPad Software, San Diego, CA, USA). Data from each animal, rather than individual sections, cells, or technical replicates, were used as independent biological replicates. For analyses based on multiple sections or repeated technical measurements from the same animal, the mean value for each animal was used as the representative value for statistical analysis. The normality of continuous variables was assessed using the Shapiro–Wilk test, and homogeneity of variance was evaluated using Brown–Forsythe’s test. Longitudinal behavioral data, including von Frey and weight-bearing test results, were analyzed using two-way repeated-measures analysis of variance followed by Tukey’s multiple-comparison test. Other continuous variables were analyzed using one-way analysis of variance followed by Tukey’s multiple-comparison test. Kellgren–Lawrence and OARSI scores were treated as ordinal data and analyzed using the Kruskal–Wallis test followed by Dunn’s multiple-comparison test. Data are presented as individual data points with mean ± standard error of the mean for continuous variables and as individual data points with median and interquartile range for ordinal scoring data, as indicated in the figure legends. A *p*-value < 0.05 was considered statistically significant.

## Results

### µCT and histological assessment

**Fig. 1:**
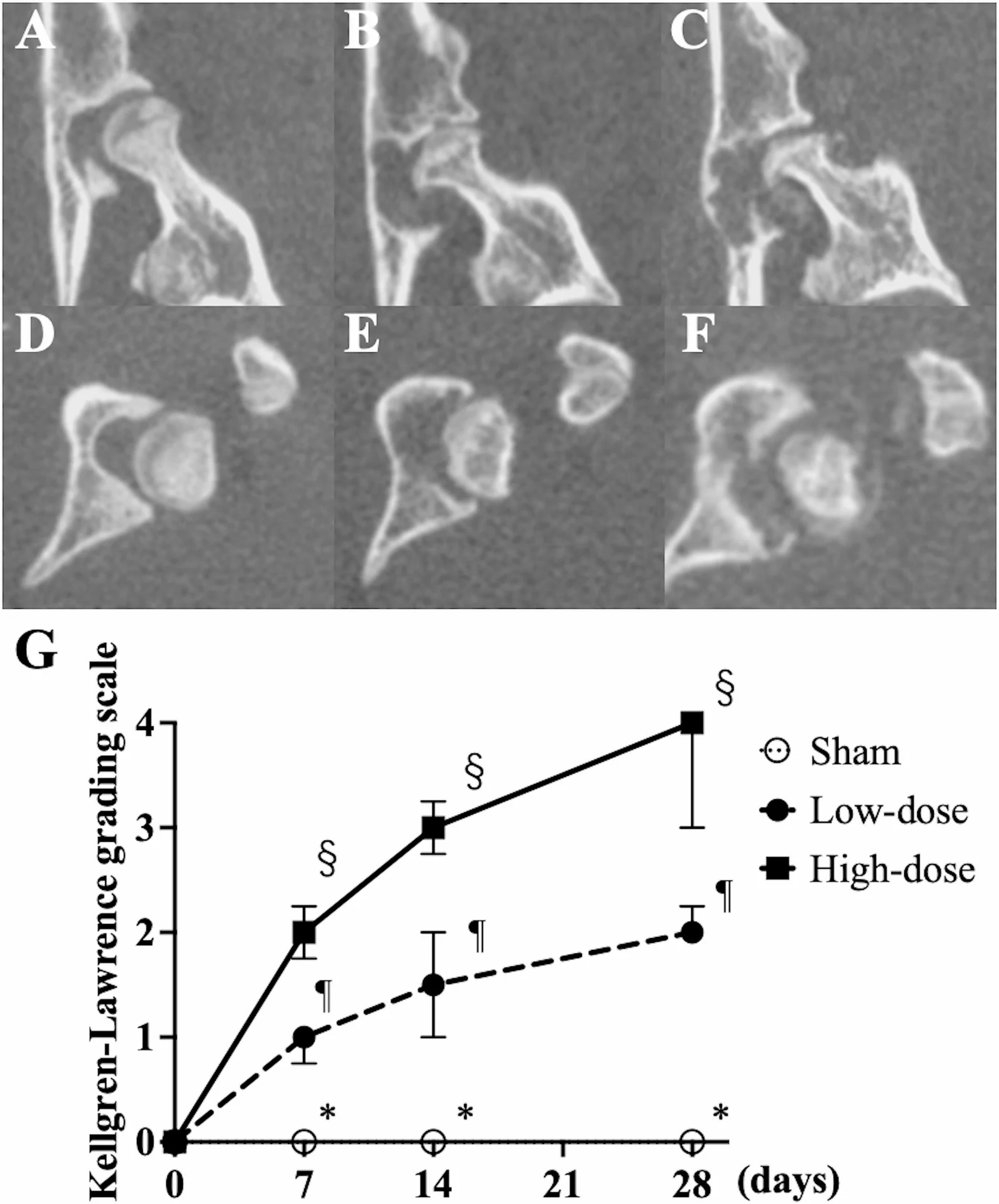
Changes in the Kellgren–Lawrence grading scale over time in each group on microcomputed tomography. Representative coronal **A**–**C** and axial **D**–**F** microcomputed tomography images were obtained immediately before and at 7, 14, and 28 days after MIA injection. The sham group demonstrated no radiographic osteoarthritic changes **A**, **D**. In contrast, both the low-dose MIA group **B**, **E** and the high-dose MIA group **C**, **F** showed progressive arthropathic changes, including joint-space narrowing and subchondral bone destruction. These changes were more pronounced in the high-dose MIA group, with marked resorption of the femoral head and acetabulum. Quantitatively, Kellgren–Lawrence scores were higher in the low-dose MIA group than in the sham group, whereas the high-dose MIA group showed significantly higher scores than both the sham and low-dose MIA groups at the post-injection time points **G**. Data are presented as the median with interquartile range; *n* = 6 per group. Statistical comparisons were performed using the Kruskal–Wallis test followed by Dunn’s multiple-comparison test. * *p* < 0.05, sham vs. high-dose MIA group. ¶ *p* < 0.05, low-dose MIA vs. high-dose MIA group. § *p* < 0.05, sham vs. low-dose MIA group

On day 28 after induction, µCT analysis revealed no apparent radiographic evidence of joint degeneration in the sham group (Fig. 1A, D). The low-dose MIA group demonstrated joint-space narrowing and subchondral bone cyst formation (Fig. 1B, E), whereas the high-dose MIA group exhibited marked joint-space narrowing with deformities of the femoral head and acetabulum (Fig. 1C, F). Quantitative evaluation using the Kellgren–Lawrence classification showed progressive radiographic joint degeneration in both MIA-treated groups. From day 7 onward, KL scores were significantly higher in the low-dose MIA group than in the sham group. The high-dose MIA group consistently showed significantly higher KL scores than the sham and low-dose MIA groups at all post-injection time points (Fig. 1G, *p* < 0.05).

**Fig. 2:**
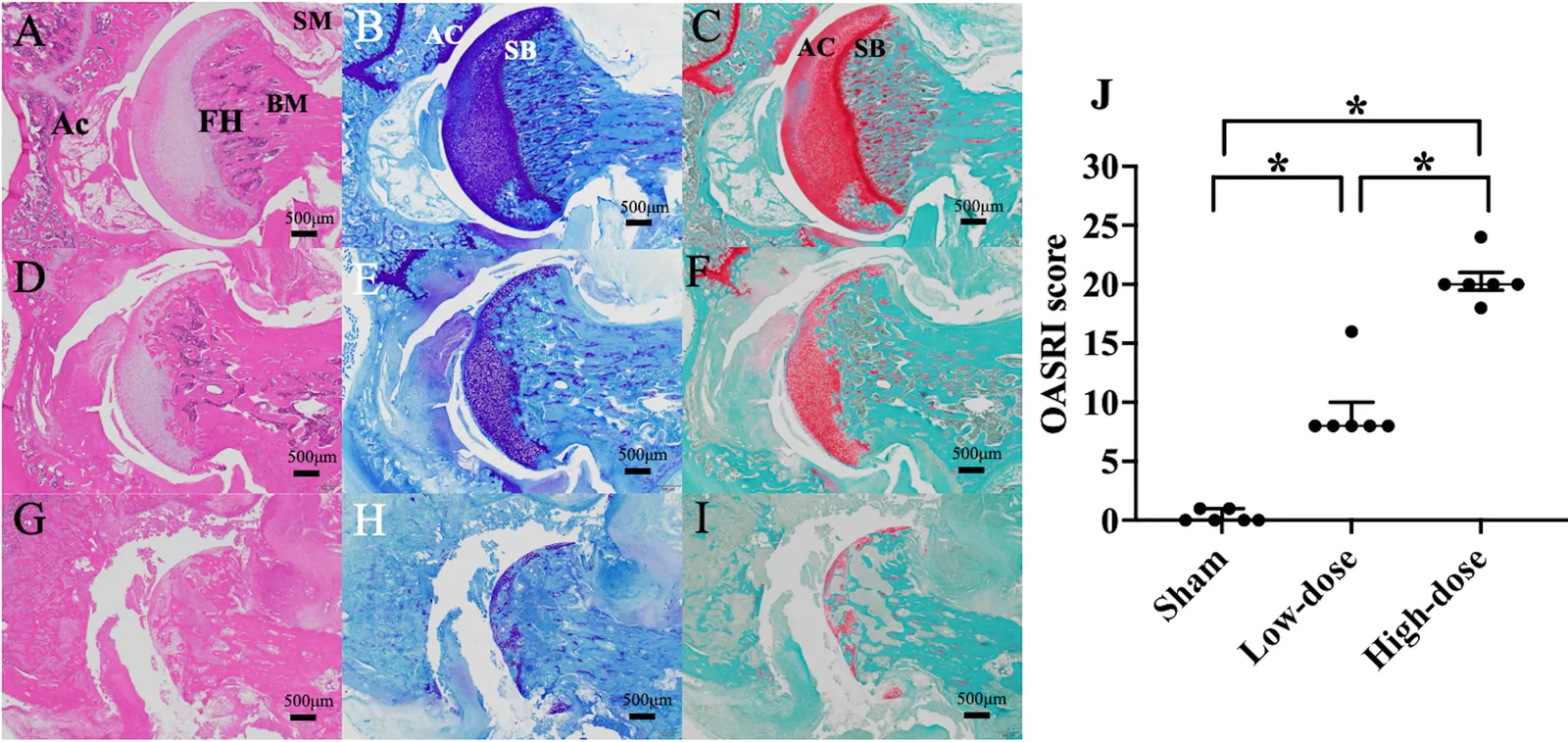
Histopathological evaluation of hip joints and Osteoarthritis Research Society International (OARSI) scoring on day 28. Representative sections of the femoral head were stained with hematoxylin and eosin **A**, **D**, **G**, toluidine blue **B**, **E,** **H**, and safranin O (**C**, **F**, **I**). The sham group showed no degenerative changes **A**–**C**. The low-dose MIA group displayed partial loss of the cartilage matrix across approximately 25%–50% of the articular surface **D**–**F**. The high-dose MIA group exhibited extensive cartilage loss with exposure and destruction of the underlying subchondral bone, affecting more than 50% of the femoral head **G**–**I**. OARSI scores **J** were significantly higher in the low-dose MIA group than in the sham group and were highest in the high-dose MIA group. Individual data points are shown with the median and interquartile range; *n* = 6 per group. Statistical comparisons were performed using the Kruskal–Wallis test followed by Dunn’s multiple-comparison test. * *p* < 0.05. Ac, acetabulum; AC, articular cartilage; SB, subchondral bone; FH, femoral head; BM, bone marrow; SM, synovial membrane

Histological analysis showed preserved articular cartilage in the sham group (Fig. 2A–C). In the low-dose MIA group, cartilage matrix loss affected approximately 25%–50% of the articular surface (Fig. 2D–F). In contrast, the high-dose MIA group demonstrated extensive exposure of subchondral bone throughout the femoral head, partly associated with fibrotic tissue formation (Fig. 2G–I). Quantitative analysis using the OARSI scoring system showed that OARSI scores were significantly higher in the high-dose MIA group than in the sham and low-dose MIA groups on day 28 after induction (Fig. 2J, *p* < 0.05), indicating more severe histological cartilage degeneration in the high-dose MIA group.

### Pain-related behavioral assessment

**Fig. 3:**
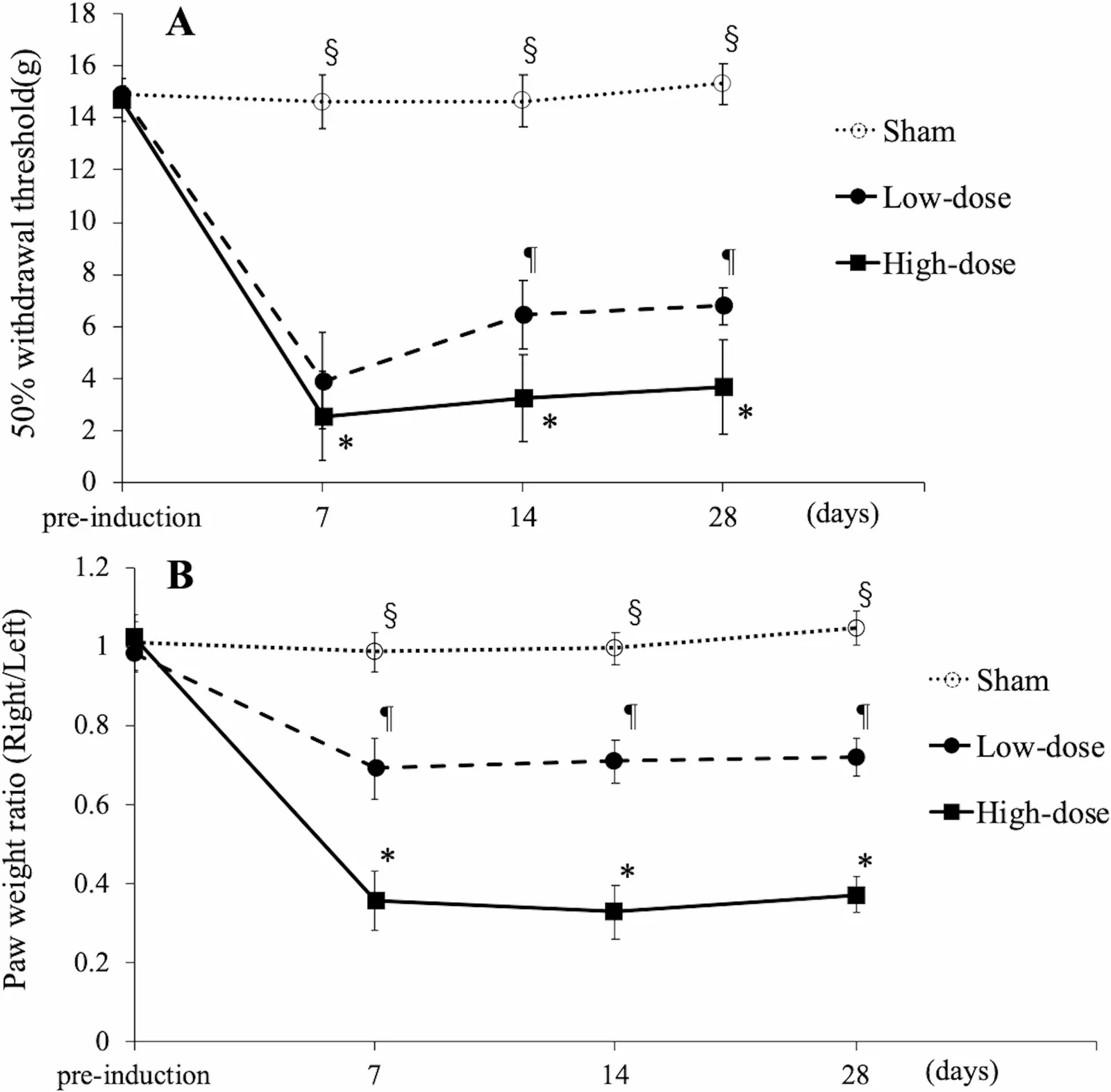
Behavioral assessment of hip pain. Mechanical hypersensitivity, expressed as the 50% withdrawal threshold, was evaluated using the von Frey test **A**. Pain-related behavior was further assessed using the static paw weight-bearing test and expressed as the weight-bearing ratio of the right limb (affected) to the left limb **B**. The 50% withdrawal thresholds and paw weight-bearing ratios were significantly decreased in the low-dose MIA and high-dose MIA groups compared with those in the sham group after MIA injection. The high-dose MIA group showed greater pain-related behavioral deficits than the low-dose MIA group, with significant differences observed on days 14 and 28 in the von Frey test and on days 7, 14, and 28 in the static paw weight-bearing test. Data are presented as the mean ± standard error of the mean; *n* = 12 per group. Statistical comparisons were performed using two-way repeated-measures analysis of variance followed by Tukey’s multiple-comparison test. * *p* < 0.05, sham vs. high-dose MIA group. ¶ *p* < 0.05, low-dose MIA vs. high-dose MIA group. § *p* < 0.05, sham vs. low-dose MIA group

From day 7 to day 28 after induction, the low-dose MIA and high-dose MIA groups showed significantly lower mechanical withdrawal thresholds than the sham group, indicating increased mechanical hypersensitivity (Fig. 3A, *p* < 0.05). The high-dose MIA group showed significantly lower withdrawal thresholds than the low-dose MIA group from day 14 to day 28. In the weight-bearing test, both MIA-treated groups demonstrated significantly reduced weight-bearing ratios on the affected limb compared with the sham group at all post-injection time points. The high-dose MIA group showed significantly lower weight-bearing ratios than the low-dose MIA group throughout the observation period (Fig. 3B, *p* < 0.05).

### Immunohistochemical analysis of the DRG and spinal cord

**Fig. 4:**
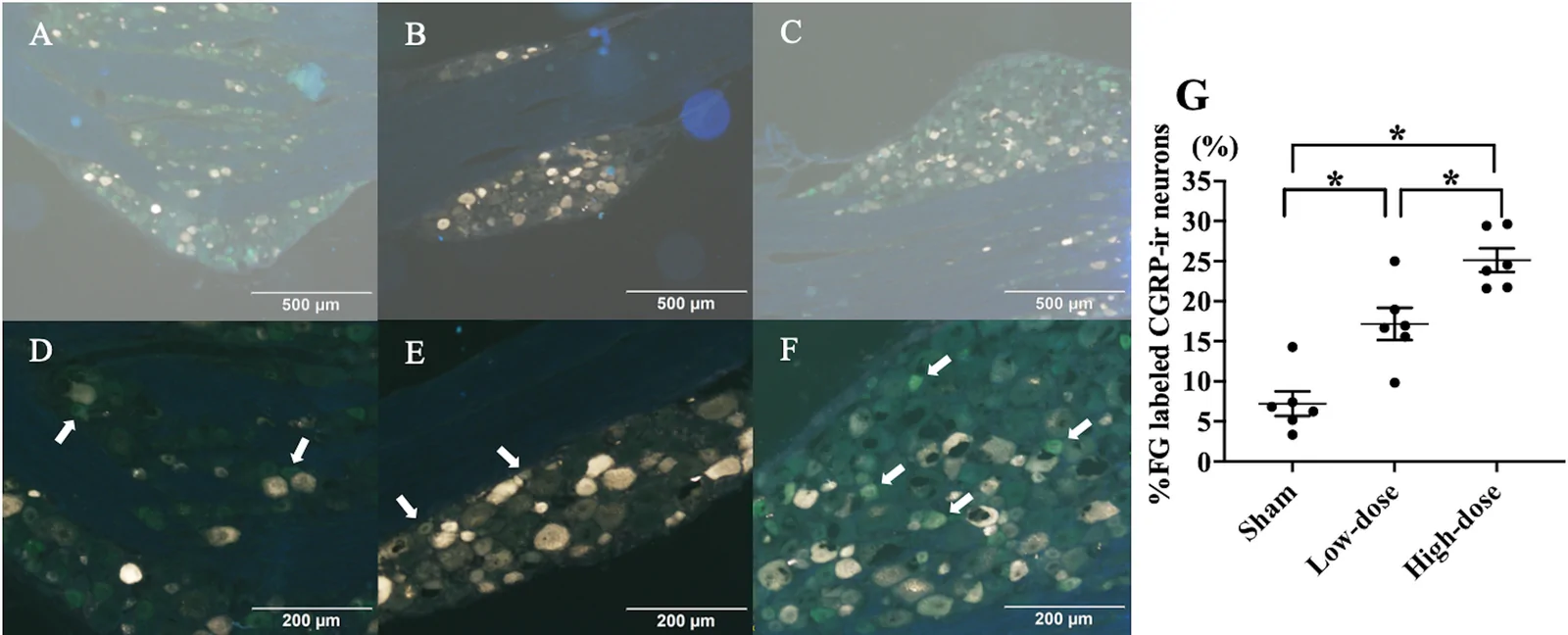
Calcitonin gene-related peptide expression in Fluorogold-labeled L4 dorsal root ganglion neurons on day 28. Representative low-magnification fluorescence images of the ipsilateral L4 dorsal root ganglion are shown for the sham **A**, low-dose MIA **B**, and high-dose MIA **C** groups. Corresponding higher-magnification images are shown in **D**–**F**, respectively. Fluorogold (FG)-labeled neurons appear gold, and calcitonin gene-related peptide (CGRP)-immunoreactive neurons appear green. Arrows indicate FG-labeled CGRP-immunoreactive neurons. The proportion of FG-labeled neurons that were immunoreactive for CGRP was significantly higher in the low-dose MIA group than in the sham group and was highest in the high-dose MIA group **G**. Individual data points are shown with the mean ± standard error of the mean; *n* = 6 per group. Statistical comparisons were performed using one-way analysis of variance followed by Tukey’s multiple-comparison test. * *p* < 0.05

**Fig. 5:**
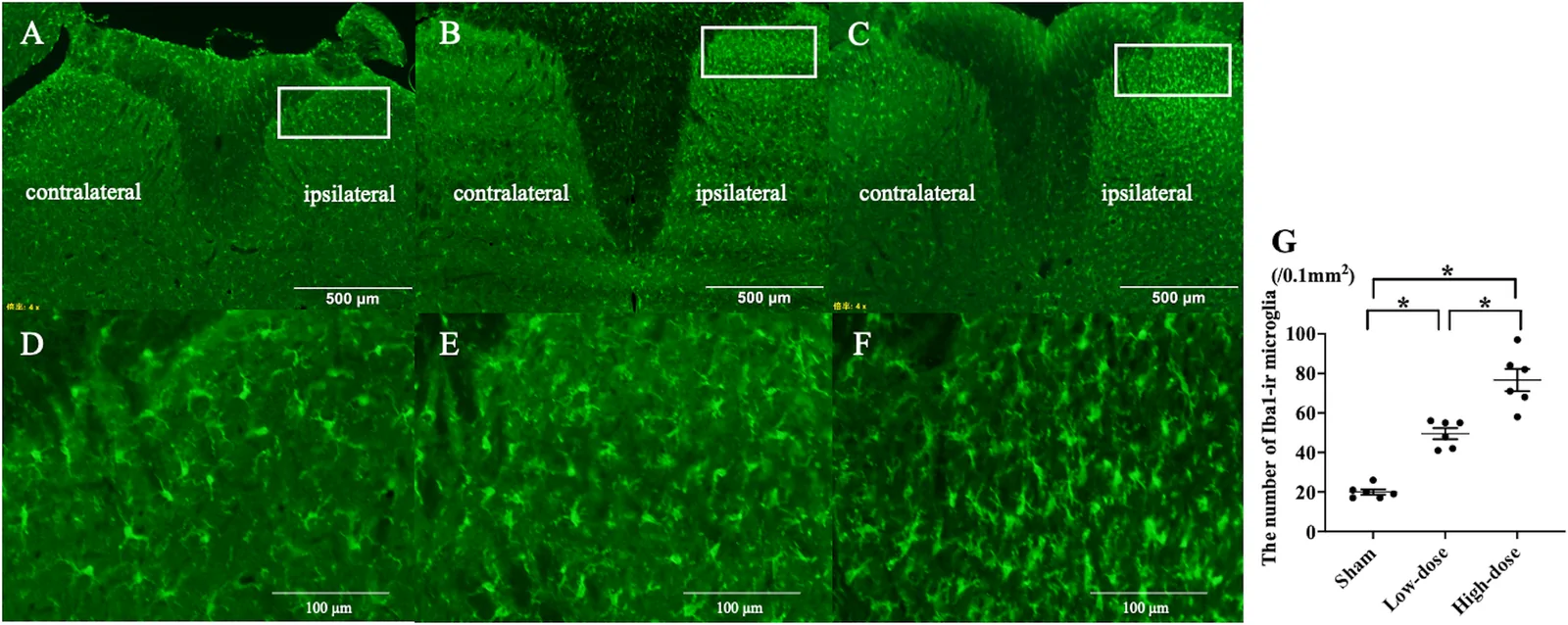
Ionized calcium-binding adaptor molecule 1–immunoreactive microglia in the lumbar spinal dorsal horn on day 28. Representative low-magnification fluorescence images of the lumbar spinal dorsal horn are shown for the sham **A**, low-dose MIA **B**, and high-dose MIA **C** groups. Corresponding higher-magnification images are shown in **D**–**F**, respectively. Ionized calcium-binding adaptor molecule 1 (Iba1)-immunoreactive microglia appear green. The number of Iba1-immunoreactive microglia per 0.1 mm² was significantly higher in the low-dose MIA group than in the sham group and was highest in the high-dose MIA group **G**. Individual data points are shown with the mean ± standard error of the mean; *n* = 6 per group. Statistical comparisons were performed using one-way analysis of variance followed by Tukey’s multiple-comparison test. * *p* < 0.05

On day 28 after induction, the proportions of FG-labeled CGRP-ir neurons in the L4 DRG were significantly higher in both the low-dose MIA and high-dose MIA groups than in the sham group. The high-dose MIA group showed significantly higher proportions of FG-labeled CGRP-ir neurons than the low-dose MIA group (Fig. 4, *p* < 0.05). Similarly, the number of Iba1-ir microglia in the spinal dorsal horn was significantly higher in both MIA-treated groups than in the sham group, with significantly greater numbers in the high-dose MIA group than in the low-dose MIA group (Fig. 5, *p* < 0.05).

### Bone-destructive environment in the femoral head

**Fig. 6:**
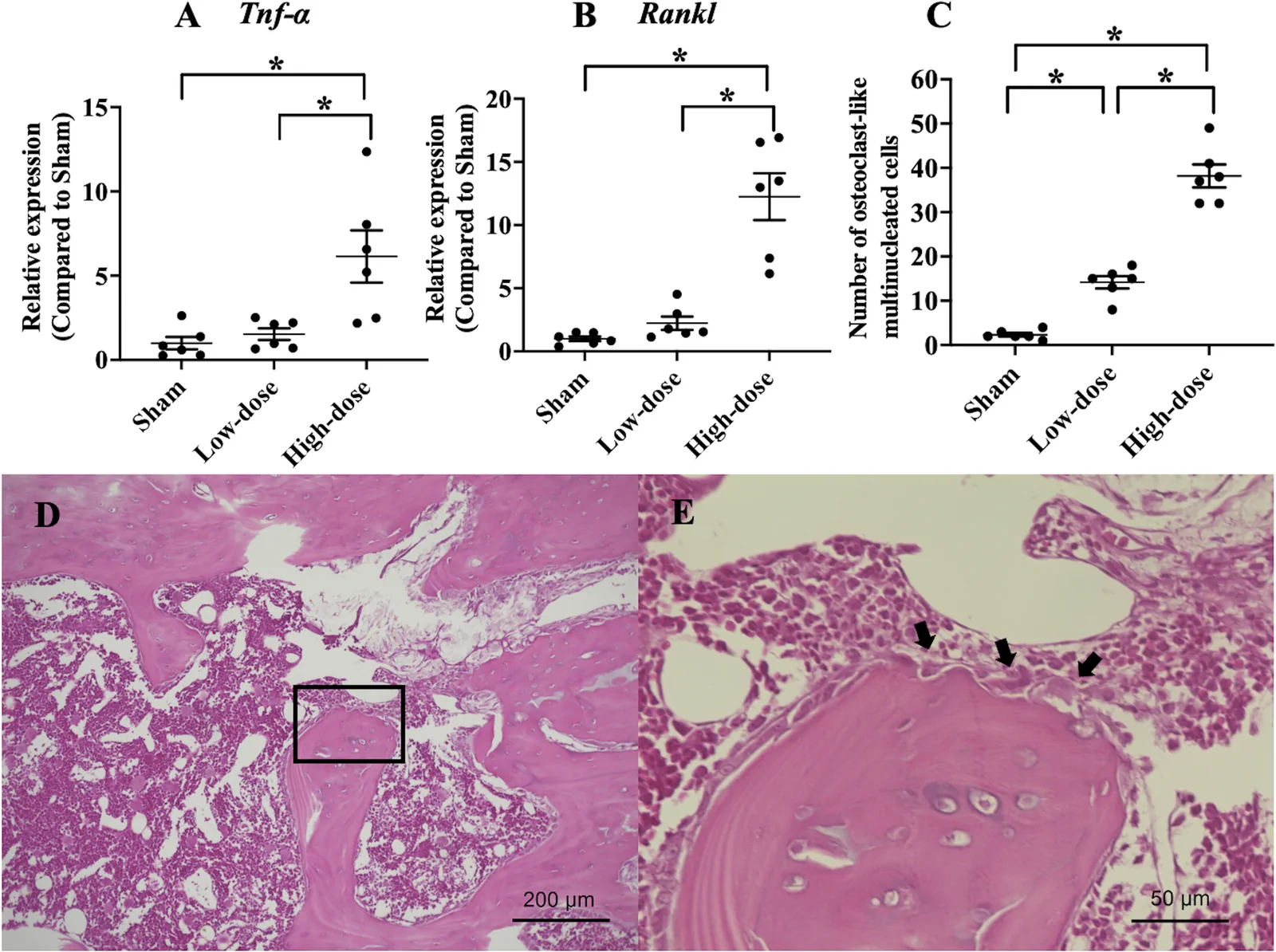
Synovial *Tnf-α* and *Rankl* expression and osteoclast-like multinucleated cells in the femoral head on day 28. **A**, **B** Relative synovial mRNA expression of tumor necrosis factor-α (*Tnf-α*) and receptor activator of nuclear factor-kappa B ligand (*Rankl*) was significantly higher in the high-dose MIA group than in the sham and low-dose MIA groups. Expression levels are presented relative to those in the sham group. **C** The number of osteoclast-like multinucleated cells in the femoral head was significantly higher in the low-dose MIA group than in the sham group and was highest in the high-dose MIA group. **D** Representative histological image of the femoral head in the high-dose MIA group. The boxed area is shown at higher magnification in E. **E** Osteoclast-like multinucleated cells in contact with the bone surface and associated with resorption cavities, including Howship’s lacunae, are shown. Individual data points are shown with the mean ± standard error of the mean; *n* = 6 per group. Statistical comparisons were performed using one-way analysis of variance followed by Tukey’s multiple-comparison test. * *p* < 0.05

On day 28 after induction, synovial mRNA expression levels of *Tnf-α* and *Rankl* were significantly higher in the high-dose MIA group than in the sham and low-dose MIA groups (Fig. 6A, B; *p* < 0.05). Quantitative histological analysis further demonstrated a significant increase in osteoclast-like multinucleated cells in the femoral head of the high-dose MIA group compared with the other groups (Fig. 6C; *p* < 0.05). In the high-dose MIA group, osteoclast-like multinucleated cells were observed along bone surfaces in association with bone resorption cavities compatible with Howship’s lacunae (Fig. 6D).

## Discussion

In this study, radiological assessment demonstrated progressive osteoarthritic changes over time in both MIA-treated groups, with more severe joint destruction in the high-dose MIA group. Femoral head flattening, joint-space narrowing, and severe bone resorption with intra-articular bone fragments occurred in the high-dose MIA group, resembling the clinical features of RDC. In human RDC, radiographic findings typically reveal rapid bone loss within months, characterized by progressive joint-space narrowing and extensive subchondral bone resorption, often without osteophyte formation—a hallmark that distinguishes RDC from ordinary OA [5]. Magnetic resonance imaging findings demonstrated femoral head flattening, cyst-like subchondral defects, and superior subluxation associated with articular cartilage loss and subchondral plate destruction [3]. Similarly, Shu et al. described intra-articular bone fragments visible on CT in patients with rapidly destructive inflammatory arthritis, suggesting that necrotic bone fragmentation and resorption contribute to the disease process [29]. Our high-dose MIA model’s radiographic features parallel these clinical findings. Histologically, at 28 days after induction, the subchondral bone of rats in the high-dose MIA group was extensively exposed throughout the femoral head and partially replaced by fibrous tissue. Numerous osteoclast-like multinucleated cells were present at the bone–fibrous tissue interface in association with resorption cavities compatible with Howship’s lacunae. These findings indicate that the high-dose MIA group developed a rapidly progressive bone-destructive phenotype with several radiological and histological features relevant to human RDC.

Compared with the sham group, both the low-dose MIA and high-dose MIA groups exhibited pain-related behaviors from day 7 after induction. The high-dose MIA group showed greater mechanical hypersensitivity than the low-dose MIA group from day 14 to day 28 and greater reductions in weight-bearing throughout the observation period. To our knowledge, this study is the first to quantitatively assess pain-related behavior in a severe MIA-induced hip arthropathy model using both the von Frey test and the WBT. Clinical data further indicate that pain is more severe in patients with RDC than in those with typical OA. Abe et al. reported significantly higher Western Ontario and McMaster Universities Osteoarthritis Index pain scores in patients with RDC than in those with OA [30]. Consistent with these clinical observations, the dose-dependent differences observed in our study suggest that the severity of joint destruction was associated with the magnitude of pain-related behavior.

To clarify the mechanisms underlying this heightened pain response, we examined CGRP expression in the DRG and microglial responses in the spinal dorsal horn. The proportion of FG-labeled CGRP-immunoreactive DRG neurons was significantly higher in both MIA-treated groups than in the sham group and was highest in the high-dose MIA group. CGRP, a 37–amino acid neuropeptide primarily localized in C-fibers and thinly myelinated Aδ-fibers, aids in nociceptive transmission and modulation [31]. Thus, the increased proportion of CGRP-immunoreactive neurons suggests enhanced peripheral nociceptive signaling with increasing severity of MIA-induced hip arthropathy. Human studies have reported that RDC synovium possesses elevated inflammatory cytokines such as TNF-α [32]. Proinflammatory cytokines directly activate DRG neurons, particularly C-fibers, by enhancing the ion channel activity and promoting neuropeptide synthesis [33], likely contributing to elevated CGRP expression and heightened pain behaviors in RDC. This interpretation is consistent with the increased synovial *Tnf-α* expression observed in the high-dose MIA group. The number of Iba1-immunoreactive microglia in the spinal dorsal horn was also significantly higher in both MIA-treated groups than in the sham group and was highest in the high-dose MIA group. Although spinal microglial changes have not been reported in RDC models, similar findings have been reported in hip and knee OA [17, 34]. Such spinal neuroimmune signaling may contribute to central sensitization and persistent pain hypersensitivity [35]. In particular, central sensitization is increasingly recognized as a contributor to persistent hip OA pain and a potential therapeutic target [36, 37]. Although increased Iba1 immunoreactivity alone does not establish central sensitization, the concomitant increases in CGRP-immunoreactive DRG neurons and spinal Iba1-immunoreactive microglia suggest that severe MIA-induced hip arthropathy involves both enhanced peripheral nociceptive input and a spinal neuroimmune response. Therefore, in addition to conventional nonsteroidal anti-inflammatory drugs, pharmacologic agents that modulate central pain pathways—such as duloxetine, which enhances descending inhibition, and pregabalin, an α2δ ligand of voltage-gated calcium channels—may warrant further investigation as potential analgesic options for severe pain associated with RDC.

At 28 days after MIA injection, the mRNA expression levels of *Tnf-α* and *Rankl* in the synovial tissue were significantly higher in the high-dose MIA group than in the sham and low-dose MIA groups, accompanied by a greater number of osteoclast-like multinucleated cells in the femoral head. Histologically, these cells were located along the bone surface in association with resorption cavities compatible with Howship’s lacunae. Because osteoclast-specific staining was not performed, their cellular identity could not be definitively confirmed; nevertheless, their morphology and localization suggest an osteoclast-associated bone-resorptive process. Clinical studies support both the inflammatory and bone-resorptive components of this interpretation. Inflammatory cytokines such as IL-1β, TNF-α, and IL-8 have been reported to accumulate in the synovial fluid of patients with RDC [12]. In parallel, Ogawa et al. reported that synovium-derived osteoclasts in patients with RDC form large lacunar resorption pits on dentine slices; such activity is absent in those from patients with OA [38]. Serological investigations have also demonstrated that compared with OA, RDC exhibits increased concentrations of markers associated with joint destruction and bone turnover, such as tartrate-resistant acid phosphatase-5b (TRACP-5b), matrix metalloproteinase-3 (MMP-3), and bone alkaline phosphatase [30], as well as a higher TRACP-5b–total P1NP ratio, indicating an accelerated, resorption-dominant pattern of bone turnover [39]. One strength of the present study is the combined evaluation of synovial *Tnf-α* and *Rankl* expression and osteoclast-like multinucleated cells across the sham, low-dose MIA, and high-dose MIA groups. TNF-α can induce RANKL expression in synovial fibroblasts and osteoblast-lineage cells, thereby promoting osteoclast differentiation [40]. At the same time, it can inhibit osteoblast differentiation, suppressing bone formation [41]. Therefore, the concomitant upregulation of *Tnf-α* and *Rankl* and accumulation of osteoclast-like multinucleated cells may contribute to the severe bone-destructive environment observed in the high-dose MIA group. Although the present observational findings do not establish causality, anti-inflammatory and antiresorptive strategies, including TNF inhibitors, bisphosphonates, and anti-RANKL antibodies, may warrant further investigation for preventing or delaying bone loss progression in patients with RDC.

Human RDC, often considered within the broader spectrum of rapidly progressive osteoarthritis (RPOA), may be better regarded as a clinicopathological syndrome arising from multiple and potentially overlapping etiologies rather than as a single disease entity. Although its pathogenesis remains incompletely understood, subchondral insufficiency fracture has been proposed as one possible initiating event leading to femoral head collapse and rapid joint destruction [13]. Mechanical factors may contribute to this process. Abnormal hip morphology, including reduced femoral head coverage, and sagittal spinopelvic changes, such as increased posterior pelvic tilt, may alter load distribution across the hip and increase focal stress on vulnerable subchondral bone [42, 43]. Early diagnosis remains challenging because subchondral insufficiency fracture and rapidly destructive hip disease may clinically and radiographically resemble osteonecrosis [43]. Moreover, pathological studies have demonstrated extensive subchondral fracture with superimposed secondary osteonecrosis, suggesting that these processes may overlap [44]. RPOA reported after anti–nerve growth factor therapy provides another important perspective. Rapid joint deterioration observed in human trials of tanezumab and after bedinvetmab treatment in companion animals has raised concern that profound analgesia, altered joint loading, and pre-existing structural vulnerability may interact to accelerate joint damage, particularly with concomitant NSAID use, although causality remains uncertain [45, 46]. These observations suggest that pain relief alone may be insufficient to address the complex pathophysiology of RDC/RPOA and highlight the need for safe disease-modifying or joint-preserving treatments. Although the high-dose MIA model does not fully reproduce the complex pathogenesis of human RDC, it provides a reproducible platform for investigating pain-related mechanisms and osteoclast-associated changes accompanying rapid hip joint destruction.

This study has several limitations. First, the low- and high-dose MIA groups represent different severities of experimentally induced hip arthropathy and should not be interpreted as direct models of conventional OA and human RDC, respectively. Moreover, the absence of a slowly progressive surgical or spontaneous OA model limits comparisons between gradual and rapid joint destruction. Nevertheless, the high-dose MIA model provides a reproducible platform for examining pain-related and bone-destructive changes accompanying severe hip joint damage. Second, the multinucleated cells observed along the bone surface were identified morphologically, and osteoclast-specific staining, such as TRAP staining, was not performed. Therefore, these cells were interpreted as osteoclast-like multinucleated cells rather than definitively identified osteoclasts. Third, quantitative µCT parameters, including femoral head bone mineral density and bone volume, were not evaluated because the original imaging protocol was designed for longitudinal morphological assessment and radiographic grading. Severe femoral head deformation and bone resorption in the high-dose MIA group also made standardized region-of-interest definition difficult. Fourth, molecular analyses focused mainly on *Tnf-α* and *Rankl*. Other inflammatory and bone-remodeling pathways, including IL-1β, IL-6, OPG, and M-CSF, as well as the roles of osteoblasts and osteocytes, were not examined. Finally, although increased *Tnf-α* and *Rankl* expression and an increased number of osteoclast-like multinucleated cells were observed, the present study was observational, and no pharmacologic interventions were performed; therefore, causal relationships among these changes and rapid bone destruction could not be established. Future studies incorporating osteoclast-specific markers, quantitative bone morphometry, and targeted pharmacologic interventions are warranted to clarify these mechanisms and evaluate potential therapeutic strategies.

## Conclusion

In conclusion, compared with low-dose MIA-induced hip arthropathy, high-dose MIA-induced hip arthropathy was characterized by more severe pain-related behavior, a higher proportion of CGRP-immunoreactive DRG neurons, an increased number of Iba1-immunoreactive microglia in the spinal dorsal horn, higher synovial *Tnf-α* and *Rankl* mRNA expression, and a more pronounced osteoclast-associated bone-destructive environment. These findings suggest that severe joint destruction in this experimental model is accompanied by concurrent peripheral nociceptive, spinal neuroimmune, and bone-destructive changes. Although it does not fully reproduce the complex pathogenesis of human RDC, the high-dose MIA model exhibits several experimental features relevant to rapidly destructive hip disease and may provide a useful platform for investigating the relationship between rapid hip joint destruction and pain-related mechanisms.

## Data Availability

The datasets generated and/or analyzed during the current study are available from the corresponding author on reasonable request.

## Abbreviations

RDC: Rapidly destructive coxarthrosis
OA: Osteoarthritis
MIA: Monoiodoacetate
µCT: Micro–computed tomography
KL: Kellgren–Lawrence
OARSI: Osteoarthritis Research Society International
DRG: Dorsal root ganglion
CGRP: Calcitonin gene–related peptide
Iba1: Ionized calcium–binding adaptor molecule 1
TNF: α–Tumor necrosis factor–α
RANKL: Receptor activator of nuclear factor–kappa B ligand
FG: Fluorogold
WBT: Weight–bearing test
qPCR: Quantitative polymerase chain reaction
